# Medical education and the quality improvement spiral: A case study from Mpumalanga, South Africa

**DOI:** 10.4102/phcfm.v7i1.738

**Published:** 2015-05-28

**Authors:** Martin Bac, Anne-Marie Bergh, Mama E. Etsane, Jannie Hugo

**Affiliations:** 1 Faculty of Health Sciences, Department of Family Medicine, University of Pretoria, South Africa; 2 MRC Unit for Maternal and Infant Health Care Strategies, Faculty of Health Sciences, University of Pretoria, South Africa

## Abstract

**Background:** The short timeframe of medical students’ rotations is not always conducive to successful, in-depth quality-improvement projects requiring a more longitudinal approach.

**Aim:** To describe the process of inducting students into a longitudinal quality-improvement project, using the topic of the Mother- and Baby-Friendly Initiative as a case study; and to explore the possible contribution of a quality-improvement project to the development of student competencies.

**Setting:** Mpumalanga clinical learning centres, where University of Pretoria medical students did their district health rotations.

**Method:** Consecutive student groups had to engage with a hospital's compliance with specific steps of the Ten Steps to Successful Breastfeeding that form the standards for the Mother- and Baby-Friendly Initiative. Primary data sources included an on-site PowerPoint group presentation (*n* = 42), a written group report (*n* = 42) and notes of individual interviews in an end-of-rotation objectively structured clinical examination station (*n* = 139).

**Results:** Activities in each rotation varied according to the needs identified through the application of the quality-improvement cycle in consultation with the local health team. The development of student competencies is described according to the roles of a medical expert in the CanMEDS framework: collaborator, health advocate, scholar, communicator, manager and professional. The exposure to the real-life situation in South African public hospitals had a great influence on many students, who also acted as catalysts for transforming practice.

**Conclusion:** Service learning and quality-improvement projects can be successfully integrated in one rotation and can contribute to the development of the different roles of a medical expert. More studies could provide insight into the potential of this approach in transforming institutions and student learning.

## Abstrait

**Education médicale et amélioration de sa qualité: une étude de cas au Mpumalanga, Afrique du Sud.**

**Contexte:** La courte durée des roulements des étudiants médicaux ne favorise pas la réussite des projets approfondis d’amélioration de la qualité qui nécessitent une approche plus longitudinale.

**Objectif:** Décrire le processus d’intronisation des étudiants dans un projet longitudinal d’amélioration de la qualité, au moyen du thème de l’Initiative des Hôpitaux amis de la Mère et des Bébés comme étude de cas; et examiner la contribution possible d’un projet d’amélioration de la qualité au développement des compétences des étudiants.

**Lieu:** Les centres de formation clinique du Mpumalanga où les étudiants médicaux de l’Université de Pretoria ont fait leurs roulements dans les districts sanitaires.

**Méthode:** Des groupes consécutifs d’étudiants ont dû, avec l’autorisation de l’hôpital, s’engager à suivre les mesures spécifiques des Dix Conditions pour le Succès de l’Allaitement maternel qui est la norme de l’Initiative des Hôpitaux amis de la Mère et des Bébés. Les sources de données primaires comprenaient une présentation PowerPoint du groupe sur le terrain (*n* = 42), un rapport écrit du groupe (*n* = 42) et les notes des entrevues individuelles dans une station d’examen Clinique structuré objectivement à la fin du roulement (*n* = 139).

**Résultats:** Les activités de chaque roulement changeaient selon les besoins identifiés par l’application du cycle d’amélioration de la qualité en consultation avec l’équipe de santé locale. Le développement des compétences des étudiants est décrit selon les rôles d’un expert médical dans le cadre du CanMEDS: collaborateur, promoteur de la santé, érudit, communicateur, gérant et professionnel. L’exposition à la situation réelle dans les hôpitaux publics sud-africains a eu une grande influence sur beaucoup d’étudiants, qui ont aussi été les catalyseurs du changement de pratique.

**Conclusion:** L’apprentissage par le service et les projets d’amélioration de la qualité peuvent être intégrés avec succès dans un roulement et peuvent contribuer au développement des différents rôles d’un expert médical. Un plus grand nombre d’études pourra donner un aperçu des possibilités de cette approche et transformer les institutions et l’apprentissage des étudiants.

## Introduction

The report of the Global Independent Commission on the Education of Health Professionals for the 21st Century recommends health education reforms that make provision for transformative learning in academic systems of hospitals and primary care networks rather than in academic centres.^1^ Universities are urged to incorporate innovative forms of learning beyond the classroom. The report states that:

[*t*]he education of health professionals in the 21st century must focus less on memorising and transmitting facts and more on promotion of the reasoning and communication skills that will enable the professional to be an effective partner, facilitator, adviser, and advocate. (p. 1945)^1^

Students should also be prepared for effective teamwork in the health system and be able to use global resources to address local priorities.^1^

Various models exist for describing the competencies of medical practitioners and how to develop them. Different sets of competencies that are applied in different countries, in undergraduate and postgraduate medical education and in different disciplines show many overlaps, with the core competencies being essentially similar. The Royal College of Physicians and Surgeons of Canada proposes the CanMEDS framework to develop leadership attributes and competencies.^2^ This model describes the different roles performed by a competent medical expert: collaborator, health advocate, scholar, communicator, manager and professional.^2^ The Accreditation Council for Graduate Medical Education (ACGME) divides the core competencies for residents into six areas: patient care and procedural skills; medical knowledge; interpersonal and communication skills; professionalism; practice-based learning and improvement (PBLI); and systems-based practice (SBP).^3^

Skills in quality improvement (QI) are regarded a necessary competency for family physicians^3,4^ and South African medical schools also recognise the importance of including QI skills in the medical curriculum for general practitioners.^5^ Medical schools in six European countries developed a framework for effective quality improvement for general practitioners and family medicine physicians based on the assumption that core QI tasks are applicable across multiple contexts, including the United States and Canada.^6^ The following domains (comprising 35 competencies) emerged from their qualitative study: patient care and safety; effectiveness and efficiency; equity and ethical practice; methods and tools; development (continuing professional education); and leadership and management.^6^

Wong et al.^7^ organised QI curricula with medical students and postgraduate trainees as targets in three main categories: formal curriculum activities for teaching concepts and methods; educational activities related to specific QI skills; and initiatives requiring participation from students. According to Van Deventer and Sondzaba, QI helps to extend students’ awareness of health systems issues beyond the traditional clinical medicine.^5^ The focus is therefore not only on clinical care, but also on issues related to organisation, ethics and patient safety.^6^

### Quality improvement in medical education at the University of Pretoria

The Department of Family Medicine at the University of Pretoria (UP) is responsible for exposing undergraduate medical students to theoretical QI concepts and methods. Students get theoretical exposure to QI at various points in the medical programme and have specific skills-development assignments to facilitate their preparation for QI initiatives. In the final 18 months of internship they are further immersed in real-life clinical practice in public hospitals and are challenged to look for opportunities to improve patient safety and patient care. During this period, students do a seven-week district health and community obstetrics rotation in a number of clinical learning centres (CLCs) attached to six district hospitals and three provincial referral hospitals in Mpumalanga Province. The purpose of this rotation is to engage them in the district health system. Students are allocated in pairs to the centres and there are usually between two and six students per CLC, with one student fulfilling the role of group leader. They also live together in the same accommodation and get to know each other socially.

One of the learning opportunities in this rotation is a compulsory QI group assignment in which students are required to be active participants in a project with a specific topic or in a specific field of study. Until 2010, students selected their own topics per rotation. The short time frame of one rotation was not conducive to the completion of all the steps in the QI cycle. Since then a more focused approach has been followed by concentrating on the same area of improvement for a longer period of time, with each group of students contributing to an on-site longitudinal QI project. A previous study of the impact of brief QI projects by medical students found that the overall outcome of a QI project was strengthened by revisiting the same topic.^5^

The objective of this article is: (a) to describe the process of inducting students into a longitudinal QI project, using the topic of the Mother- and Baby-Friendly Initiative (MBFI) as case study; and (b) to explore the possible contribution of a QI project to the development of student competencies.

## Research methods and design

### Ethical considerations

Ethical approval for studying this approach to QI was obtained from the Research Ethics Committee of the UP's Faculty of Health Sciences (S160/2009) and the Provincial Health and Research Ethics Committee of the Mpumalanga Department of Health. The purpose of the study was explained to students during their orientation for the rotation.

### Choice of focus and study period

For 2012, the topic selected for the students’ QI projects was infant feeding, with special attention on hospital activities to become designated as mother- and baby-friendly, according to the World Health Organization (WHO)/UNICEF guidelines.^8^ The choice of this topic followed on previous student activities related to the integration of maternal and child health services^9,10,11,12^ and as a response to South Africa's poor performance on the achievement of the Millennium Development Goals (MDGs) 4 and 5.^13^ A further impetus was the August 2011 Tshwane Declaration of Support for Breastfeeding in South Africa, which called for the accreditation – by 2015 – of all public hospitals and health facilities according to the standards of the MBFI.^14^ A number of the hospitals where students do rotations had lost their mother- and baby-friendly status in the recent past, mostly as a result of not fully complying with steps 1 (written breastfeeding policy), 2 (training of all staff) and 10 (mother support groups). Furthermore, in April 2012, a national policy directive regarding the implementation of the 2010 WHO prevention of mother-to-child transmission (PMTCT) guidelines came into effect and the provision of free formula on demand for HIV-exposed infants was stopped.^15^

The MBFI QI programme was conducted in Mpumalanga Province over a period of one year (2012), with a follow-up study in the last rotation of 2013. The study included 139 students in six rotations and nine clinical learning centres in 2012 and 32 students in eight CLCs in the last rotation of 2013.

### The educational learning experience

**FIGURE 1:**
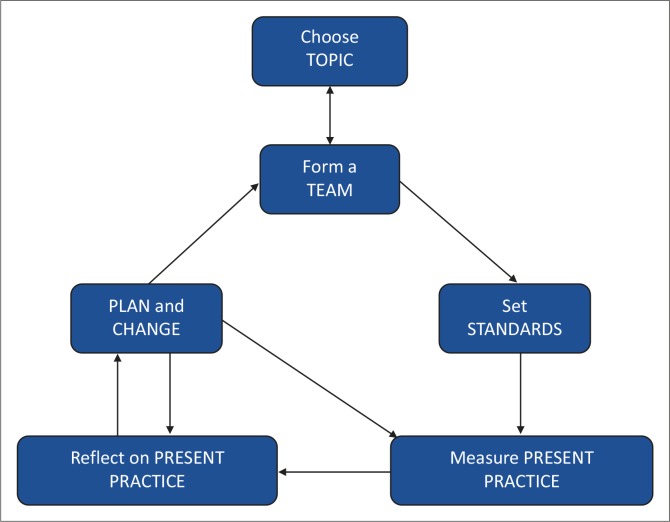
The quality improvement cycle used for the student projects.^16^

**FIGURE 2:**
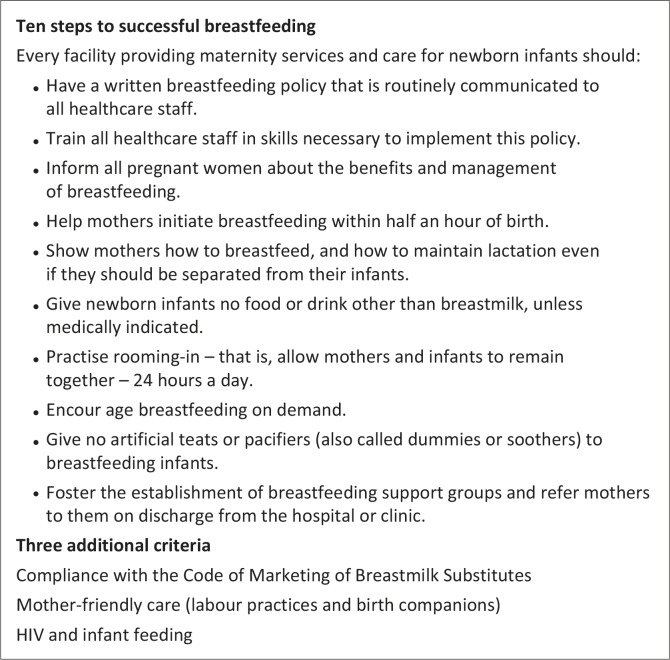
Standards for mother- and baby-friendly hospitals.^8^

Students received face-to-face orientation and a written guideline on their project. They were also taken through the QI cycle (Figure 1) to illustrate how their assignment featured as part of a spiral. They were instructed to form a team with relevant role players. The team ideally included their on-site mentor and key personnel in the hospital, clinic and/or sub-district (dieticians, nurses, midwives, doctors and managers). For the MBFI, the standards had already been set in the form of the 10 steps to successful breastfeeding and the three additional criteria derived for South Africa from the revised international guidelines for the Baby-Friendly Hospital Initiative (BFHI) (see Figure 2).^8^ Students’ main function was to assess present practice and improvement in the adherence to the national and international standards. By reflecting on present practice they were then expected to make recommendations to address the detected discrepancies between the standard practice and current practice. The next group would then be required to follow up on the implementation of recommendations and work on further improvement:

‘Our project was part of the “Plan and Change” step of the QI cycle. The groups before us had already identified the standards and measured the current practices in place and so it was our role to implement change using their insight as our driver.’ (HospH Rot3)

In the first rotation, students started out by exploring the reasons for the hospitals’ loss of mother- and baby-friendly status, where applicable, and other problems related to infant feeding. Students focused on the first step that pertains to the facility's breastfeeding policy. In the second rotation, the focus was on training (step 2). In all rotations, students had to observe skin-to-skin practices in the labour ward and report on progress. In some rotations, students did infant-feeding surveys by means of standard questionnaires and interview guides provided by the university. In the last rotation of 2012 and of 2013, students had to interview health workers on their views regarding breastfeeding policies, how policy changes affected their work, what they considered major infant-feeding challenges and recommendations with regard to the latest infant-feeding policy.

For the first two rotations, the guidelines were fairly uniform but started to diversify per centre as each QI project developed a ‘life of its own’, determined by the context and staff needs. From the second rotation onwards, students also received copies of the reports from previous groups for the purpose of continuity and to build on work already done. One example of project diversification was Hospital A, where students could not make headway with the work required for steps 1 and 2 and, as a result, went on to step 10 by assisting the establishment of breastfeeding support groups in the community.

### Data sources and analysis

Specific requirements for student groups and individuals at the end of each rotation included an on-site group presentation in PowerPoint, a written group report and a six-minute individual interview at a station of the end-of-rotation objectively structured clinical examination (OSCE) (with an interviewer and a note taker). These documents were the primary data sources for our analysis. In 2012, a total of 139 students participated in the OSCE interviews. There were 34 reports and presentations in 2012 and eight in the single rotation in 2013. Three hospitals had students for all six rotations in 2012; two had students for four rotations; two for three rotations; and two for one rotation only.

Three researchers studied the reports and presentations before each OSCE. Thereafter, two researchers immersed themselves in the reading and re-reading of the reports to identify the different roles performed by students according to the CanMEDS framework. OSCE notes were then checked for any insights that could complement the results from the analysis of the reports and presentations.

Trustworthiness^17^ was pursued by means of triangulation of data sources (different sites; different rotations) and utilising two data analysts. Credibility was further enhanced by the group report and the verification of individual students’ involvement in the QI project in the OSCE interviews. Rich descriptions in the form of direct quotations from students’ reports and references to interviews indicate the authentic voices of individual participants and groups and allow for transferability and confirmability. The detailed description of the educational learning experience should enable other researchers to conduct a similar study and contributes to dependability.

## Results

### Student roles and competencies

We used the CanMEDS framework to describe the ideal of the different roles students had to perform in developing their competency to conduct QI inquiries and to reflect on the extent to which students were able to practise these competencies. The QI project contributed to the development of students as *medical experts*. The MBFI is a patient-centred initiative, which enabled students to become health advocates and integrate their knowledge, clinical skills, procedural skills and professional attitudes linked with a public health perspective and non-clinical roles.^5^ The focus was therefore on bridging the gap between evidence, policy and practice.^4^ With the limited resources available, students had to come up with feasible proposals to manage and improve patient care within the specific context where they worked. Students also had to learn how to collaborate and use their personal communication skills in a scholarly manner within the constraints of the public health system.

#### Collaborator

The role of the collaborator is to work effectively ‘within a healthcare team to achieve optimal patient care’ (p. 5).^2^ Teamwork was the essence of the QI projects:

‘We took the role of members of the quality improvement team … We couldn’t have achieved our goal without the help and support of our various team members and are thankful for the role they play in our, but also in the patient's lives. We aim to work together as a great team to achieve ultimate Baby Friendly Status.’ (HospA Rot5)

The challenge students faced was working across groups of professionals in different sections of the hospital, such as the labour and postnatal wards and the neonatal unit. They also had to collaborate with dieticians, quality assurance officers and managers. In some rotations, student QI activities extended to adjacent clinics, schools and communities:

‘We didn’t encounter much [*sic*] obstacles, everyone worked together very well. The only thing that was a bit of a problem was the clinics’ involvement in the breastfeeding policy … The clinics are functioning very well individually, but they must realise what more they could achieve by working as the bigger A [*town name*] team …’ (HospA Rot2)

Although students were required to act as collaborators, they experienced professional boundaries and hierarchies as important obstacles in the smooth functioning of interprofessional activities – ‘We have arranged a meeting in the hope of having as many multidisciplinary attending as possible; unfortunately only the dieticians attended’ (HospA Rot3). For some centres, students described their reception as welcoming and inclusive – ‘Upon arrival at Clinic Z [*a community health centre*] we were greeted as old friends and we felt warm in our hearts’ (HospH Rot4). At Hospital H, students also became ex-officio members of the breastfeeding committee working towards regaining the hospital's mother- and baby-friendly status. At other centres, students felt they had a hostile reception and struggled to form a team with the health professionals; *inter alia* as a result of strained interprofessional relationships:

‘We followed the steps of the cycle and found some of the shortcomings to be: lack of staff motivation, no multidisciplinary involvement, inadequate communication, overall apathy towards breastfeeding importance and lack of commitment.’ (HospG Rot1)

Similarly, students sometimes found the good attendance of their end-of-rotation presentation by staff members ‘overwhelming’ (OSCE HospH Rot2), whereas in other instances ‘attendance of the presentation was poorer than expected. … We had hoped that the Maternity Staff would attend, and we were rather shocked that not one was present’ (HospA Rot2).

#### Health advocate

The health-advocacy role entailed the students’ immersing themselves in the national and international literature and taking this vision to the hospital where they were placed. Despite staff ignorance and resistance, they had to play the role of advocates on behalf of mothers and babies to make the health services more mother and baby friendly. One of their first activities was to determine the current status of the hospital's breastfeeding policy (step 1). In the majority of the hospitals that had lost their mother- and baby-friendly status, the existing policy had been found inadequate. Students’ advocacy role included assistance with up-dating the policy or continuous enquiry into the state of policy development where there had been no or slow progress over time:

‘We first consulted with the labour ward matron concerning their policy of a baby friendly hospital and the implementation of this policy. There is, in fact, a policy in the hospital, but we found it has not been strictly adhered to and poorly implemented. The policy is in fact outdated and the BFHI committee is in the process of reviewing and writing up a new policy meeting the standards set for the BFHI. We reviewed the 10 steps to successful breastfeeding and noted the points of concern for hospital H and where they need to work on and improve.’ (HospH Rot1)

Students also had the role of enquiring about the activities of the breastfeeding committee, which was defunct in a number of the hospitals:

‘We were lucky to have the chance to attend the first two meetings of a new committee that included the dieticians for the first time, the postnatal staff and paediatric staff.’ (HospA Rot1)

‘It was found that the last proper training session and documentation of breastfeed training at Provincial Hospital B took place in 2006. This was obviously not sufficient and an investigation to get to the root of the problem was launched.’ (HospB Rot5)

At individual sites, a number of other advocacy roles were observed. As part of their project students had to enquire about and observe skin-to-skin practices immediately after birth during all rotations. At some sites they had to lead by example when doing their own deliveries to illustrate that skin-to-skin contact was feasible, even if they had not always been able to change midwifery practices in general:

‘With each patient we managed, we were successful in counselling and initiating both breastfeeding and skin-to-skin care. After practising it ourselves we informed the nursing staff that it was possible. In spite of this, the beliefs of the staff have remained unchanged.’ (HospG Rot3)

In one instance, students discovered that free formula was still being issued by the hospital after the introduction of the new infant feeding policy on 01 April 2012. After the matter had been reported, the practice was stopped.

#### Scholar

The scholarly activities of students included reading up about the MBFI and breastfeeding, as well as doing small surveys on infant-feeding intentions and/or practices in pregnancy, the immediate post-partum period and at postnatal follow up between six weeks and six months after birth:

‘To accomplish these goals [*setting up a support group, developing a breastfeeding counselling checklist*] we had to consult literature concerning skin-to-skin care, breastfeeding and quality improvement methods from sources such as the WHO, Medline and Cochrane. This data was utilised to design the breastfeeding pamphlet, develop our literature review and support some [*of*] our recommendations.’ (HospA Rot4)

Students had to disseminate their findings and recommendations at the end of each rotation in a presentation and report to local management, the breastfeeding team and other stakeholders. In this way they had to transform the literature reviewed and the data collected into new knowledge that could be applied to the local situation. Scholarly activities had an influence on both students and healthcare workers:

‘We also found that the babies aren’t placed on the mother's chest at all until they reach Post Natal Ward, thus revealing that skin[*-to-skin*] care is not really practised … This raised awareness in us as the advantages of skin to skin care are numerous … and therefore emphasis should be placed on promoting skin-to-skin care.’ (HospA Rot5)

‘Sr. Sithole* did not have a written guideline for the exact method of performing adequate skin to skin contact and we therefore undertook a literature review to seek answers. Our plan was mainly aimed at educating healthcare workers in the district.’ (HospD Rot5) (Pseudonym*)

#### Communicator

In the guidelines that the students received at the beginning of the rotation, the importance of good communication with the local facilitator and other key role players was emphasised in order to establish rapport and trust. In their project reports, students were also required to give feedback on their consultation process with the health team. Intra-group communication amongst students had to be facilitated by the group leader in order to have good collaborative learning:

‘Our group leader was exceptionally proficient and guided the group with no difficulty. Whilst being steady and fair, he was also accommodating and considerate. He skilfully directed the project and assigned tasks with seemingly no effort, masterfully considering the strengths and capabilities of each team member. This ensured that the best possible result was produced in the shortest amount of time.’ (HospH 2013)

Delivering information and reaching a mutual understanding are important components of good communication. In this regard students acted as informants, educators and trainers. At some sites they did their compulsory skills-training session on MBFI-related topics. They were also involved in staff training sessions that were part of some hospitals’ MBFI training (step 2). Good communication skills were required in cases where students met with resistance and conflict within the health team. Students also learned about poor intra- and inter-institutional communication in the health system that led to inertia with regard to training and the inability to involve clinics in the MBFI:

‘One very noted point that was received with a lot of anger and agreement [*during the presentation*] was the one pertaining to shifting of [*training*] responsibility [*between labour ward and postnatal ward staff*]. It was the one point of total agreement and has been promised to be rectified.’ (HospB 2013)

‘We have noted in discussions with both members of the breastfeeding committee and in a Subdistrict PHC [*primary healthcare*] unit managers meeting, that there is a degree of blameshifting between the two parties [*hospital and clinics*].’ (HospA 2013)

#### Manager

The QI project demanded from students the use of different skills as managers. Decision making was a golden thread throughout. Students had to schedule and harmonise their individual and group activities with the other requirements and compulsory activities within the rotation. This required good time management and leadership in public health settings where these qualities are often lacking. Some students commented on the unexpected workload accompanying the QI project, which posed a challenge to individuals to complete their tasks and to the group to prioritise appropriately:

‘Our Quality Improvement Report took longer than expected to write up but we are proud of the end result. We did not anticipate how much time it required, perhaps we would have started sooner if we had some foresight.’ (HospD Rot5)

Visiting different clinics to collect data for the surveys required logistical coordination. Data capturing and analysis had to be organised and responsibilities allocated. In the case of poster and pamphlet development, printing and distribution of material had to be costed and managed:

‘As we identified that individual “Feeding the Future” cards are not economically feasible, we opted for larger posters to be prominently displayed in the relevant wards and clinics. Time will be set aside to educate sisters of the above mentioned clinics on the importance of the “Feeding the Future” poster and how they can use it to educate mothers on breastfeeding at every antenatal care visit.’ (HospH Rot4)

The ability to bring all the threads together at the end of the rotation into a coherent presentation and report demonstrated a group's ability to execute tasks collaboratively. The group report had to list individual student contributions and in the OSCE students were probed in such a way that the issue(s) discussed would demonstrate identification with and intimate involvement in the project as a whole. Only one student demonstrated involvement of less than 5/10.

#### Professional

In the MBFI QI project, students expanded their role as professionals by discovering that the art of medicine is much more than the care for individual patients:

‘While assessing what the focus of our QI project would be, we reviewed the quality improvement cycle and the principles of quality improvement in district hospitals. In doing this we decided to take three main mantras to heart, being: Focus on the patient; Start small; and Seek solutions.’ (HospA Rot3)

During their project, students not only became more knowledgeable about breastfeeding and the importance of sound infant-feeding practices, but also developed realism and professional insight in matters related to public health and provider and client behavior:

‘We are of the view that if the focus is shifted from regaining status to making an impact on the lives of the mothers and children passing through the hospital and clinic system, it would foster the environment that MBFI aims to achieve.’ (HospA 2013)

‘One of the mothers unfortunately didn’t receive the breastfeeding message with similar enthusiasm due to her HIV status. She had read in the package insert … contraindicated to breastfeed … she was unyielding to change her decision. This was an important lesson for all the group members as we realised the impact of HIV related stigma. This type of stigma may be more deeply entrenched in the community than we at first thought.’ (HospH Rot3)

### Achievements, challenges and limitations

It was not possible to measure the effect of students’ involvement in QI quantitatively in terms of organisational changes or patient outcomes, as the projects were embedded in the specific context of a complex healthcare setting, sometimes with many underlying problems where students landed in the proverbial cross-fire of different hospital factions:

‘The presentation initiated a heated discussion between the staff from the labour ward, postnatal ward, family medicine practitioners, dieticians and the matron. This discussion culminated in the formation of a new approach to the QIP for the next group.’ (HospG Rot4)

Students at two sites concluded at the end of 2012 that little progress had been made with the MBFI:

‘It seems that from the start of this extended quality improvement little progress has been made with the BFHI.’ (HospB Rot5)

On the other hand, many students commented on the meaningfulness of their experience, even in hospitals where MBFI progress was perceived to be slow or absent – ‘You can make a difference in a place like this’ (OSCE HospG Rot3).

‘Despite the challenges we faced we are proud of the work we produced and the contributions we made to Hospital D. We are grateful for the learning experience!’ (Hosp D Rot5)

‘“That's one small step for man, one giant leap for mankind.” It was a great privilege to play even a minor role in such a massive worldwide venture. We realised that even a small group of people can make a massive difference.’ (HospA Rot5)

Students were exposed to the planning and implementation of change and, although they were mostly not champions leading change, they demonstrated the possibility of being temporary change agents^3^ or catalysts working together with local change agents in one team – ‘During our … rotation we met a lot of passionate people, which is the most important ingredient to success’ (HospA Rot5).

‘In attendance [*at our presentation*] was the head matron, whom it seemed we reached with our presentation … The matron was the only person out of all that attended that felt like she could make a difference from here on and that, after we’ve left, she can continue with all the previous groups’ and our efforts … Overall we were happy with the reaction to our presentation, though most of it was negative, a discussion did ensued [*sic*] and critical thinking processes were started.’ (HospA Rot6)

Although not generalisable, students contributed to the MBFI by working on the shortcomings identified in previous internal and external hospital assessments – ‘Ultimately our progress as a group lay in the realisation that the previous groups have instilled a good backbone for skin-to-skin support and [*MBFI*] implementation’ (HospA 2013).

‘The breastfeeding committee expressed a great amount of gratitude as it relates to the contributions of the previous group[*s*] in 2012. They explained that the majority of the input was made in the relevant wards, where practices were changed significantly. They also explained that the hospital's breastfeeding committee received extremely helpful recommendations from this group of students.’ (HospH 2013)

Students’ influence and contribution were visible in a variety of activities and events – ‘We made headway in policy approval, reforming a committee, organising staff training, educating staff at the rural clinics and initiating patient education’ (HospG Rot3).

Probably the most rewarding for students was the independent reaccreditation of two institutions in 2012 and the continued role they could play in the MBFI in 2013:

‘There is no better way to summarise what we have been trying to do at Hospital H than to acknowledge the reinstatement of their ‘Baby-Friendly’ status as of November 2012. The endorsement of the External Provincial Assessment report clearly shows that the interventions focussed upon by this year's students in breastfeeding and skin-to-skin care have made a positive and lasting impact for Hospital H and its patients and employees.’ (HospH Rot3)

‘We became part of the team as the men (and women) on the ground. We assisted with the monitoring and observation of the implementation of the policies put in place in labour ward and post natal ward. We were good for the role as the staff carried on with their duties without altering them for our sake as compared to if any of the MBFI members were to walk in. The other role we played was to evaluate the real circumstances and how possible it is to implement all the policies in the real situations in the wards. This made us realise that students form an invaluable part of the MBFI committee even though each group is present in the hospital for only six weeks, especially in short-staffed hospitals like Hospital F.’ (HospF 2013)

Other achievements included the following:

- Revival of and participation in breastfeeding committees (albeit with variations in lasting results).
- Attendance of other decision-making meetings.
- Participating in internal assessments at the request of a breastfeeding committee or a nursing manager.
- Update of policies.
- Inputs in the initiation and/or overhaul of training programmes.
- Development of educational materials (3 breastfeeding pamphlets, 3 posters, 1 ‘Feeding the Future’ motivational card for mothers, 2 breastfeeding counselling checklists).
- Active participation in breastfeeding week/month activities (3 sites).

‘The most positive change that we noticed was the fact that more counselling on breastfeeding was taking place now.’ (HospB 2013)

### Three important lessons

#### Top-down themes and topics

The organisation of the QI projects for the MBFI was an attempt by the university to align with recent government developments to promote the achievement of MDG 4. For the programme coordinators, the ability to guide students in a more uniform manner was attractive. However, this approach led to diverse responses at hospital level, with some hospitals embracing student participation and others showing little or no interest. In the welcoming hospitals there was a direct need to prepare for reaccreditation in 2012 and 2013.

‘For us to be given the opportunity to present our case and findings for people as respected and high up as the head matron … and the CEO [*chief executive officer*] of the hospital show how serious Hospital H is about regaining this BFHI recognition. We contributed on various levels of the quality improvement cycle and process.’ (HospH Rot1)

‘The only problem with the implementation of the 10 steps with regards to the committee is that the committee only reassures the implementation of the 10 steps when there is an evaluation and it is not constantly checked upon.’ (HospD 2013)

#### Ownership of quality improvement

Through the student reports we observed different degrees of hospital ownership of the MBFI – ‘No ownership of projects’ (OSCE HospA Rot 2). The fact that the topic of MBFI was chosen by the university in consultation with the provincial health department might have influenced ownership of QI positively or negatively. Students as ‘outsiders’ bringing a topic not selected by the local hospital appeared to be less welcome in certain hospitals – ‘Matron forgets meeting appointment’ (OSCE HospG Rot 2). This illustrates a challenge of bridging the policy-practice gap in hospitals lagging behind in their MBFI efforts. The selection of local topics related to specific public health policies, in consultation with the on-site university facilitator and other key personnel, may improve local ownership and involvement of students in an on-going QI programme of the hospital.

‘The other major problem is the lack of an active chairperson to lead the breastfeeding committee. No-one is involved to ensure continuous progress monitoring or the implementation of trainings, meetings, etc. We raised this topic with the dieticians who agreed with our observation and decided that as it was an internal matter is [*sic*] should be dealt with internally.’ (HospA Rot3)

#### Short versus longitudinal projects

Six rotations on the same theme were too long for some of the hospitals and we observed fluctuating enthusiasm amongst the student groups and ‘topic fatigue’ from the on-site health team toward the end.

‘We got the impression that they [*clinic staff*] are either tired of students interfering and would lie to get rid of us or that they are scared that we would report them to some authority for not being up to the required standard.’ (HospA Rot6)

For 2013, hospitals also identified shorter QI projects on a broad range of topics. Having a mix of short- and long-term QI projects (single or multiple QI cycles and/or spirals) can improve the ownership and responsibility of the local health team to implement the recommendations originating from the student projects. This mix has already shown potential for more intensive collaboration between students and the health team in projects for the improvement of pain control during labour and modifiable factors contributing to maternal mortality.

## Discussion

The study of final year medical students’ longitudinal QI projects demonstrated that these projects could be part of their normal curriculum for developing their non-clinical competencies. Students acted as triggers to facilitate the implementation of new policies in the form of the MBFI guidelines and to narrow the gap between policy and practice. For many students this was a first-time experience in hospitals outside metropolitan areas and they gained new perspective on the role doctors should be able to play. Vildbrad and Lyhne^18^ suggest that non-medical expert roles are often neglected in the medical curriculum and that medical schools should create opportunities for students to develop these competencies. QI projects, especially those related to system-based practice, should be integrated in the normal curriculum in medical schools^19^ and should not only be on a voluntary basis for those with an interest.

When applied to the ACGME core competencies, most of our students’ activities were related to PBLI and SBP.^3^ These competencies contribute to empowering ‘learners with the skills to plan, lead, and execute health care systems improvement efforts’ (p. 93).^20^ There is evidence that the study participants developed and practised these skills successfully and with enthusiasm, thereby fulfilling their professional role. The phrase ‘making a difference’ was frequently used to describe QI project experiences. This is similar to the outcomes of a Joint Royal College of Physician Training Board project that demonstrated the ability of trainees to lead small-scale change that can contribute to improving multidisciplinary teamwork, clinical practice and patient care.^21^ The positive student experiences reflected a sense of student group ownership,^22^ even if there were QI ownership issues at some of the learning sites.

Choudhery et al.^23^ identified five practical areas to make QI projects valuable learning experiences: more awareness of QI and ideas for projects; promoting faculty mentorship and publication; education on project design and implementation; resources (e.g. books and funds); and dedicated time allowed. Vaux^21^ refers to an enabling infrastructure to be able to make a difference. Our QI programme in an under-resourced provincial public health system was made possible by the infrastructure provided through the CLCs and the on-site mentors who received sufficient information to further guide and support students during their rotation. Guidance students received on the areas of MBFI to address in their activities included aspects of design and implementation. Students received resources in the form of academic and website references and had access to the university library, although the focus was more on teamwork and sharing their learning with the health workers on site.^24^ As part of their compulsory activities, students had to spend time on their QI activities, and this provided a unique opportunity to familiarise students and hospital staff with new policy developments and translate them to local protocols. The longitudinal nature of the QI programme provided for a longer time frame to complete the individual site projects and allowed for continuity in learning experiences. The interdisciplinary nature of this programme and the intensive group work challenged students to excel in utilising and further developing their non-clinical skills.

Varkey and Karlapudi^20^ refer to programme experiences on the challenges to implement PBLI and SBP curricula because of lack of time and resources and a perception that PBLI and SBP are not relevant to future careers. Our students found challenges in the following forms: resistance to change practice (especially skin-to-skin care); interpersonal tensions; slow progress on the uptake of student recommendations in some of the learning centres with the implementation of certain of the 10 steps to successful breastfeeding; high workload as a result of staff shortages; insufficient continuity of staff in management positions and in membership of breastfeeding committees; poor interdisciplinary collaboration; and lack of integration between extra- and intra-mural health services. Some of these challenges are similar to the findings of a study by Watts et al.^25^ that identified the following major challenges: staff turnover; competing priorities; no clear sense of focus; no link to performance assessment; no clear sense of added value; inability to translate provided tools into viable projects; and incomplete teams. The authors stated that sustainable, continuous QI initiatives needed more than dedicated leadership support, formal education sessions and dedicated non-clinical time.

Research limitations were the relatively small number of hospitals participating, the single focus on MBFI and the time period that did not allow for drawing conclusions on the long-term impact. Students do not have the authority and power to implement their recommendations and even with a longitudinal QI approach this remains a major limitation in achieving significant change in some hospitals. Studies are needed on how this type of experiential service learning contributes to transformational learning in order to be able to make recommendations to other medical schools with regard to longitudinal approaches to QI in medical education. Further studies on the ideal mix between single and longitudinal QI projects may provide more insights into improving the service-learning experience of students and how to improve the local ownership. Whereas the current study focused on the student experiences of their QI projects, views of health care providers on the longitudinal QI approach should also be elicited more systematically. Notice should also be taken of other maternal and child health interventions promoted by current initiatives such as the Priority Cost Effective Lessons for System Strengthening South Africa (PRICELESS SA).^26^

## Conclusion

This study illustrates how QI projects carried out in a platform of hospitals contributed to the development of the non-medical competencies of a medical expert, especially where there is a clear focus, explicit instruction and adequate mentorship. The experience with the MBFI QI projects illustrated how the third QI curriculum category requiring active student participation^7^ can be implemented in a meaningful manner. Students were required to initiate projects in consultation with the health team and to be the ‘conscience’ in cases where progress with the implementation of the MBFI steps was slow. The exposure to the real-life situation in South African public hospitals had a great influence on many students as expressed in their personal reflections on the rotation. In addition to contributing to the development of their competencies, it could be argued that the QI projects transformed the students as well as the institutions. Turning individual QI projects into a longitudinal QI programme may have contributed to sustain some of the changes achieved.
